# Employees and the National Insurance Act

**Published:** 1912-04-27

**Authors:** 


### NATIONAL INSURANCE ACT DEPARTMENT.

### EMPLOYEES AND THE NATIONAL INSURANCE ACT.

### VIII.—The Contributions Payable.

There does not yet appear to be any widespread enthusiasm amongst workers generally over the National Insurance Act. In spite of lectures delivered by the hundred, and leaflets issued by the million, there is still a large amount of popular indifference to the scheme. Much of this is doubtless due to the fact that the benefits conferred by the Act are still a long way off, but perhaps even to a greater extent it is occasioned by the complicated nature of its provisions and the difficulty of realising the advantages of health insurance in an abstract form. But the time will come when the possibilities of the Act will be appreciated more readily than they are at present.

In this department of The Hospital we are treating the Act as it stands, without political bias of any description, in an endeavour to explain to our readers the benefits provided for them, and with a view to assisting them in matters of doubt and perplexity. The general provisions of the Act in regard to benefits have already been dealt with, though we shall have to return to this topic as occasion demands when referring to the application of the Act to special classes of persons and in special circumstances as to age, remuneration, and arrears of contribution.

Contributions.

In this issue we deal with the general subject of the contributions required—how, when, and by whom they are payable, and the rates applicable to employer and employee respectively. Though the benefits receivable may seem to be long deferred by reason of the waiting periods, the time for the commencement of payment of contributions is at no great distance. On Monday, July 15 next, according to present arrangements, the Act is to come into operation, and on that day the first contributions will become due. It has been commonly stated that the employee, the employer, and the State all contribute their quota. This is literally true, but there is a fundamental difference between the contributions of the employee and employer on the one hand and the contribution of the State on the other hand. The former are payable weekly, or at other prescribed intervals, at rates specified in the Act, whereas the latter take the form, not of additions to the contributions, but to the benefits payable, including the cost of administration. The proportion of the funds, out of which the benefits and the cost of administering them are payable, to be provided by Parliament, is in the case of men two-ninths and in the case of women one-quarter. In order to illustrate this statement it may be said that for each 7s. worth of benefit provided by the joint contributions of employer and employee in the case of men the State will add a further 2s., and will, in addition, pay the like proportion of the expenses of administration. For each 6s. payable as benefit to, or on behalf of, a female contributor the addition of the State will also be 2s. This is not quite the same thing as saying that for each 7d. contributed by, and in respect of, an employed male, or for each 6d. in the case of a female, the State will add a further 2d. The distinction is rather fine, but the point will be readily grasped when it is remembered that the benefits do not become payable until after certain qualifying periods have elapsed, and in the case of sickness benefit, for example, at least twenty-six weekly contributions have been paid. Small though the distinction may be, it will give rise to a considerable saving to the Treasury in the current financial year, and for some time to come the Government grants will be less than they would have been if they had been payable as a proportion of the contributions instead of as additions to benefit.

Rates of Contribution.

The rates of contribution are set out in the Second Schedule to the Act. This schedule is divided into two parts, the first of which is applicable to insured persons in Great Britain, and the second to those resident in Ireland. The reason for this differentiation lies in the fact that medical benefit is not included in the list of benefits obtainable in Ireland, and, in consequence, the Irish rate of contribution is less than that payable in either England, Scotland, or Wales.

Dealing first with the rates of contribution in Great Britain, the weekly sum payable on account of each employed male contributor will be 7d. In the case of women the weekly contribution is 6d. Of these sums the standard contribution payable by the employer is 3d. per week, the balance of 4d. per week, if the insured person is a man, or of 3d. per week in the case of a woman, having to be found by the insured person. There are, however, certain exceptions to the proportions in which the contributions are allocated to the employer and the employee respectively. These exceptions depend upon the rate of remuneration, and in calculating such rate consideration is given to the provision of board and lodging by the employer. When no such provision is made and the employed contributor, of either sex, is at least twenty-one years of age and in receipt of a wage which does not exceed 1s. 6d. per working day, the contributor is relieved of all personal contributions. In such case the sum of 1d. per week is to be made available out of moneys provided by Parliament, and the balance of the contribution—namely, 6d. in the case of men and 5d. in the case of women—will be charged to the employer. Under similar conditions, except that the remuneration exceeds 1s. 6d. but does not exceed 2s. a working day, the State grant remains the same—

namely, 1d. per week, but the contributor will be charged 1d. per week, reducing the charge, as compared with the previous case, against the employer to 5d. per week for men and 4d. per week for women.

When the rate of remuneration exceeds 2s. but does not exceed 2s. 6d. per working day, the State grant of 1d. per week is not given, and in the case of women workers the distribution of the charge is as in the normal standard—namely, 3d. each per week against the employer and employed. If the insured contributor is a man his contribution will be 3d. per week and that of the employer 4d., thus reversing the proportions as set out for the normal standard.

### Low Wages.

The special treatment of low-paid labour is intended to discourage the payment of sweating wages, and to throw the cost of protecting the health of the worker on the employer. One point that should be particularly noted is that the special schemes only operate when the worker is twenty-one years of age or upwards, so that the benefit of the provision does not apply to insured persons between the ages of sixteen and twenty-one. It is thought in some quarters that this will lead to an increase in the employment of young persons at the expense of their elders in certain occupations; but it is difficult to see that it can have any such effect. The extra charge on the employer, as his contribution under the Act, for adult labour over that required for persons under twenty-one when low rates of wages are paid is at its maximum only 3d. per week, and this will, in most cases, be more than set off by the larger output of the experienced hand.

### Irish Rates of Contribution.

The full rates of contribution applicable to insured persons in Ireland are 1½d. per week less than those for Great Britain—that is to say, the rates are 5½d. a week for men and 4½d. for women. Of this 1½d. the employer receives the benefit of ½d. and the contributor obtains the reduction of 1d. Similar arrangements to those already described apply in the case of low rates of remuneration, and we need not enter into a detailed statement of the various proportions in which the reduction is divided between the employer and contributor.

We have referred to the contributions of the employer and of the employed person, but though the total sum can be so divided it is in essence one contribution only. The two parts are not payable independently of each other. In the first instance the employer is responsible for the payment of the contributor's share as well as his own, but he is given power to recover the amount paid on behalf of the contributor by deduction from wages or otherwise.

The rules governing the payment and recovery of contributions are set out in the Third Schedule to the Act, which comprises eleven sub-sections.

### Rules Governing Payment.

These rules provide that for each calendar week—which, for the purpose of the Act, means the period from midnight on one Sunday to midnight on the following Sunday—during the whole or any part of which an employed contributor has been employed the full weekly contribution shall become payable. This means that if only one day's work is done in a week the contribution payable is not that for one day only, but the full rate for the week. If services have been rendered to more than one employer during the same week only the one contribution will be payable. No contribution is, however, to be payable by an employer for any week during which no services have been rendered and no remuneration paid, or when no services have been rendered and the insured person has been at any time in that week in receipt of sickness or disablement benefit. The first of these exemptions from payment covers the case of a holiday extending for a full week, when no remuneration is paid for such period.

### Exemption from Payment: An Example.

In the event of a holiday being granted and the remuneration being continued though no service is rendered, it would seem that the contribution must be paid as usual. The second exemption is designed so that the sickness or disablement allowance shall be paid in full without deduction for the current weekly contribution. It is to be noted, however, that the contribution is apparently payable unless no services have been rendered during the week. Thus, for example, if the insured contributor does a day's work on a Monday and falls ill on Tuesday, the incapacity for work lasting up to and including the following Thursday week, the sickness benefit will commence on the Friday, but as he did a day's work on the preceding Monday a week's contribution will be payable. Having recovered before the end of the second week, the two days' work in that week will require the payment of the contribution for that week. It will thus be seen that, although a full week's sick pay will have accrued, the contributions will in such circumstances be payable without remission. In more extended sickness attacks, the advantage to the insured person of receiving the benefit free of deduction will be considerable, for, though the contribution is small and may not be missed when in receipt of full wages, the deduction of 3d. or 4d., as the case may be, from the allowance, particularly when the disablement rate of 5s. a week is in operation, is a matter of no small consideration.

We shall continue the subject of the contributions in our next issue.

NOTE:—Questions and Correspondence on all doubtful points are invited, and should be addressed to the Insurance Editor, The Hospital, 28 Southampton Street, Strand, W.C.

### Answers to Correspondents.

"D. B., Maidstone": Yours is a very hard case. When the Act comes into force in July you would be wise to join a benefit society, and we will make inquiry for you which would be the best under your circumstances. In all probability the pension you already receive will be made up to £13 a year, under the head of Disablement Allowance. You have our sincere sympathy. Let us know if you would like some one to call on you. Address Editor of The Hospital, Bureau of Information, 28 Southampton Street, Strand, W.C.

